# P-544. Whole Food Plant-Based Diet for HIV-Associated Reduction in Cardiovascular Risk: A Pilot Randomized Clinical Trial (PLANT-HART)

**DOI:** 10.1093/ofid/ofae631.743

**Published:** 2025-01-29

**Authors:** Carolina Martinez-Loya, Ruth Selene Favela Ortiz, Alejandra Vargas-Castañeda, Mariana Seijas-Vasquez, Jesus Perez-Castilla

**Affiliations:** Universidad Autónoma de Chihuahua, Chihuahua, Chihuahua, Mexico; Universidad Autónoma de Chihuahua, Chihuahua, Chihuahua, Mexico; Universidad Privada San Juan Bautista, Ica, Ica, Peru; Universidad Nacional Romulo Gallegos, Elizabeth city, North Carolina; Universidad Nacional de San Antonio Abad del Cusco, Cusco, Cusco, Peru

## Abstract

**Background:**

Globally, 39 million people are affected by HIV, most of whom are on antiretroviral therapy (ART), which increases cardiovascular risks. Guidelines lack specific dietary recommendations for this population, though plant-based diets may reduce these risks. This pilot study (NCT05796882), to our knowledge, is the first to explore the effects of a plant-based diet on cardiovascular risk in HIV-positive individuals.

**Table 1.**
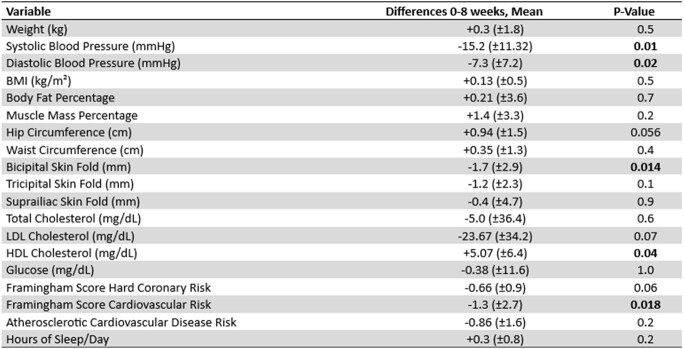
Differences in variables for both groups combined and their statistical significance. Paired sample analysis.

**Methods:**

This randomized controlled pilot clinical trial, conducted in Mexico, involved HIV-positive adults on stable ART, aged 30-60, with a body mass index > 25 and ≥ 1 cardiovascular risk factor. Exclusion criteria included: acquired immunodeficiency syndrome, major cardiovascular events, substance abuse, and ART non-adherence. Of 150 invited, 21 consented and 10 completed the 8-week intervention. Outcomes were analyzed using non-parametric tests (Wilcoxon Sign-rank and Mann-Whitney U). The ethics committee approved; all participants provided informed consent.

**Results:**

Ten male participants (mean age 40.3 ± 8.2) were divided into a plant-based diet (PBD, n=4) and a nutritional standard care (NSC, n=6) group. After 8 weeks, reductions in cardiovascular risk were observed; the NSC group experienced a -0.9% in Framingham Hard Coronary Risk (p=0.2) and the PBD group a -0.1% (p=0.1). Blood pressure decreased significantly in both groups: systolic blood pressure decreased by 15.2 mmHg (p=0.01) and diastolic blood pressure by -7.3 mmHg (p=0.02) (Table 1). The PBD group also showed reductions in weight (-700g, p=0.7), body fat percentage (-0.9%, p=1.0), tricipital fold (-3mm, p=0.06), total cholesterol (-32.0 mg/dL, p=0.1) and LDL cholesterol (-39.7 mg/dL, p=0.2), though not statistically significant.

**Conclusion:**

This pilot study suggests that a plant-based diet may reduce cardiovascular risks in HIV-positive individuals. Both groups were significant for reducing systolic and diastolic blood pressure. The plant-based diet can potentially be beneficial for weight and cholesterol management. Despite a small sample size and adherence challenges, regular follow-ups and dietary self-reports enhanced compliance, underscoring the need for larger, comprehensive studies to confirm these findings.

**Disclosures:**

**All Authors**: No reported disclosures

